# How can forest management increase biomass accumulation and CO_2_ sequestration? A case study on beech forests in Hesse, Germany

**DOI:** 10.1186/s13021-019-0132-x

**Published:** 2019-12-17

**Authors:** Joachim H. A. Krug

**Affiliations:** Silviculture and Forest Ecology of the Temperate Zones, Georg-August-Universität Göttingen, Büsgenweg 1, 37077 Göttingen, Germany

**Keywords:** Human-induced management impact, Cumulative biomass growth, Forest growth, *Fagus sylvatica* L., Growth-dominance, Factoring out

## Abstract

**Background:**

While the capability of forests to sequester carbon dioxide (CO_2_) is acknowledged as an important component in fighting climate change, a closer look reveals the difficulties in determining the actual contribution by forest management when indirect and natural impacts are to be factored out. The goal of this study is to determine the direct human-induced impacts on forest growth by cumulative biomass growth and resulting structural changes, exemplified for a dominating forest species *Fagus sylvatica* L. in central Europe. In 1988, forest reserves with directly adjacent forest management areas (under business as usual management) were established in the federal state of Hesse, Germany. Thereof, 212 ha of forest reserve and 224 ha of management area were selected for this study. Biomass changes were recorded for a time span of 19 to 24 years by methods used in the National Inventory Report (NIR) and structural changes by standard approaches, as well as by a growth-dominance model.

**Results:**

The results indicate a higher rate of cumulative biomass production in the investigated management areas and age classes. The cumulative biomass growth reveals a superior periodic biomass accumulation of about 16%. For beech alone, it is noted to be about 19% higher in management areas than in forest reserves. When harvests are not included, forest reserves provide about 40% more biomass than management areas. The analysis of growth-dominance structures indicates that forest management led to a situation where trees of all sizes contributed to biomass increment more proportionally; a related increase in productivity may be explained by potentially improved resource-use efficiency.

**Conclusions:**

The results allow a conclusion on management-induced structural changes and their impact on carbon sequestration for *Fagus sylvatica* L., the dominating forest species in central Germany. This affirms a potential superiority of managed forests to forests where the management was abandoned in terms of biomass accumulation and reveal the impact and effect of the respective interventions. Especially the analysis of growth-dominance structures indicates that forest management resulted in more balanced dominance structures, and these in higher individual biomass increment. Forest management obviously led to a situation where trees of all sizes contributed to biomass increment more proportionally.

## Background

The capacity of forests to sequester carbon dioxide (CO_2_) is gaining more attention in political and public discussions. Since the global CO_2_ level is continuously rising, enhancement of CO_2_ sequestration by forests seems to be an important component in fighting climate change, but proposed approaches differ fundamentally. While, e.g., the German forest owner’s association, supported by Prime Minister Laschet, proposes to remunerate forest owners for CO_2_ sequestration [1, 2], contradicting voices recommend protecting forests against sustainable management as a contribution for climate protection [3, 4].

One important background of such opposing approvals is the challenge to identify the contribution of forest management on enhanced CO_2_ sequestration. Despite common beliefs, it is difficult to determine the impact of management practices on forest growth, since it requires a differentiation between natural, indirect, and direct human-induced impacts on forest growth. This is especially difficult in uneven-aged forest stands. On top of this, common yield tables tend to be outdated since additional impacts, like an enhanced CO_2_ concentration, nitrogen (N) depositions, and changed precipitation and temperature patterns, have initiated increased growth rates [5–7].

The challenge to identify the human-induced impact on forest growth gained specific importance in climate change negotiations, when it was decided that CO_2_ sequestration by forests can be accounted for and be used to compensate for emissions from other sectors [8, 9]. Thus, to enable an accounting approach for forest management, it is necessary to distinguish between “direct human-induced contributions” and “natural” or “indirect human-induced contributions” to the overall C stock change and GHG emissions of forest ecosystems [9]. The Marrakesh Accords decision 11/CP.7 on Land Use, Land-Use Change and Forestry (LULUCF) invited the Intergovernmental Panel on Climate Change (IPCC) in 2001 to “develop practicable methodologies to factor out direct human-induced changes in C stocks and greenhouse gas emissions by sources and removals by sinks from changes in C stocks and greenhouse gas emissions by sources and removals by sinks due to indirect human-induced and natural effects (such as those from carbon dioxide (CO_2_) fertilization and nitrogen (N) deposition), and effects due to past practices in forests (pre-reference year), to be submitted to the Conference of the Parties at its 10th session” [10]. However, the IPCC meeting on “current scientific understanding of the processes affecting terrestrial carbon stocks and human influences upon them” concluded in 2003 that the “scientific community cannot currently provide a practicable methodology that would factor out direct human-induced effects from indirect human-induced and natural effects for any broad range of LULUCF activities and circumstances” [11].

Also, the “managed land proxy”, the assumption to exclude natural and indirect human-induced contributions by only considering managed lands, and related approaches of the Paris Agreements could not provide a solution on that challenge [12, 13].

Current scientific approaches to estimate the impact of direct human-induced management made use of models, e.g., based on carbon flux measurements [14–17], or were based on comparisons between old-growth forests with permanent forest estates, e.g.[18, 19]. However, such comparisons may suffer appropriateness since not only site conditions but also stand structures are of limited comparability and lack comparable system boundaries. When, for example, the focus is set on the net primary production assessed by carbon flux measurements, harvests as a distinct part of net primary production are ignored, which may cause misleading results, e.g., [4, 20]. In a recent publication, Herbst et al*.* presented a comprehensive study on net atmospheric carbon dioxide exchange, total evaporation, and net primary production of two neighbouring beech forests in central Germany [18]. One of these forests (individual ages between 0 and 250 years) has remained without major management impacts since 1965 (besides an extraction of single, very valuable trees), while the second, an even-aged (about 130 years old) managed forest, was kept under business as usual (BAU). It was found that the average carbon fluxes measured over seven years did not differ significantly between the two forests. Such results might be based on different stocks of the two compared sites.

Besides methodological challenges, there is an on-going debate whether or not it would be more effective for climate change mitigation not only to manage but to allow forests to grow without management for increased and/or additional carbon sequestration [19–21].

While model projections of various management scenarios, including the abandonment of thinning regimes, wood extraction etc., based on general growth assumptions in central Europe exist [22–26], “real data” representing a database sufficient to allow general statements is scarce [21]. More critically, Nabuurs et al*.* concluded by comparing uncertainties in carbon sequestration estimates for a tropical and a temperate forest, “even in a case with good access to data, the uncertainty remains very high, much higher than what can reasonably be achieved in carbon sequestration through changes in forest management” [27]. Further, most discussions do not differentiate between direct human-induced and other human-induced impacts, like increased concentrations of CO_2_ or increased N deposition, a prolonged vegetation period, or changed precipitation rates. Those indirect human-induced impacts also lead to increased forest growth but cannot be attributed to forest management practices, as the impacts affect managed as well as unmanaged forest [6]. Since it is quite difficult, if possible at all, to determine the effect of such “other” human-induced impacts than management, only a direct comparison of managed and unmanaged forests can allow a conclusion on the impact of management on superior biomass development and, from this, the impact on carbon sequestration.

Comparisons of average carbon flux measurements also provide valuable results on the magnitude of carbon sequestration [19, 28]. However, such are of little help to access the actual impact of forest management as long as no more detailed insight is delivered on the specific processes – unless managed and unmanaged forests are directly compared. To provide a better understanding on the impact of management practices, a more “clear cause and effect relationship between management practices and carbon stocks in different compartments of the ecosystem is required” [29].

To overcome the above-mentioned limitations, the research objective of this study is to evaluate management impacts on cumulative beech biomass growth, respectively carbon sequestration. For this, managed and directly neighbouring, unmanaged forests are compared. In the following, the term “unmanaged” reflects the management decision to abandon wood extraction, thinning measures etc. It is a specific goal to investigate the relation between increment changes and management impacts on the stands’ structures. For this, management-induced changes on growth dominance structures (determined as a function of stem mass in relation to stem mass increment) are investigated. Such changes are expected to reduce competition pressure and are assessed to analyse respective differences of productivity.

Limitations are set by the focus on biomass production, which is related to carbon sequestration. Other forest management goals are ignored without prejudgement of respective importance. Also, past treatment of the stands influences growth and biomass accumulation after abandonment of wood extraction. Therefore, only pairwise comparisons with directly neighbouring stands of common management history are to be considered. It is also assumed that the pairwise comparison eliminates the need to consider additional influencing factors relevant to growth beyond management impacts for the purpose of this study.

Further on, it must be considered that the forest reserves, although unmanaged for about 30 years, cannot be compared to natural forest or forests’ structures with old-growth attributes. The comparison between managed and unmanaged sites can only serve as an analysis of changes within the observed time period and age classes. Structural investigations of natural and old-growth forest are provided, e.g., by [30–32].

The study focuses on one specific forest management type within a regional restriction. Nevertheless, the database represents the dominating forest type for central Germany; the direct comparison of comparable management and non-management forest sites in such instances is rare in central Europe.

## Results

### Statistic evaluation

The statistical evaluation allows the assumption that changes in living biomass between the first and second inventory can be related to different management intensities. Biomass changes within the sites (forest reserves and management areas) and within the two inventory years were evaluated statistically by ANOVA analysis and a post-hoc-test (linear hypothesis contrast test). The normal distribution was tested by the Kolmogorov–Smirnov test. All cases where H_0_ = data are normally distributed were rejected. The results of the analysis of living biomass between forest reserves and management areas in the first and second inventory year, as well as the different forest reserves and management areas between both inventory years, are illustrated in Table 1.

**Table 1:** Statistical analysis of living biomass between forest reserves (FR) and adjacent neighbouring management areas (MA) in the first and second inventory year as well as of the different FR and MA between both inventory years (ANOVA and Post-Hoc-Test)

Site	ANOVA	Post-Hoc-test			
		FR: first to second inventory	MA: first to second inventory	First inventory: FR to MA	Second inventory: FR to MA
802 Goldbach	R^2^ = 0.0267 F(3.3,103) = 28.42 P < 0.001	P = 0.250	P = 0.058	P < 0.001	P < 0.001
803 Schönbuche	R^2^ = 0.2193 F(3.2,961) = 277.05 P < 0.001	P < 0.560	P < 0.001	P = 0.005	P < 0.001
808 Hohestein	R^2^ = 0.0607 F(3.5,196) = 111.91 P < 0.001	P < 0.001	P < 0.001	P = 0.249	P < 0.001
809 Haasenblick	R^2^ = 0.0520 F(3.5,239) = 95.72 P < 0.001	P < 0.001	P < 0.001	P = 0.091	P < 0.001
827 Weserhänge	R^2^ = 0.0734 F(3.3,417) = 90.26 P < 0.001	P < 0.001	P < 0.001	P < 0.001	P = 0.436

### Human-induced changes in biomass, increment and dominance structures

#### Management impacts on biomass and increment

The stands’ biomass accumulation and increment within different age classes differ significantly between the managed and unmanaged stands. As a result, managed stands provide lower basal areas in combination with higher increment rates in almost all age classes and a higher biomass accumulation of about 16% than unmanaged stands.

First, common to all five sites is a closed-canopy structure at the time of the first inventory, with some understory of natural regeneration, while beech represents about 93.7% (± 5.8%) of the stands’ biomass on average. The dominating layer of each site depicts an age range of less than ± 10 years at the time of the first inventory on average, attributed to former management approaches favouring even-aged stands. Due to non-management and management-induced changes in the age structure, all sites reflect a more multi-layered and uneven-aged structure at the time of the second inventory.

Second, although the stands reflect very different diameters and volume distributions when compared among each other, the impact of management is clearly visible.

**Fig. 1:**
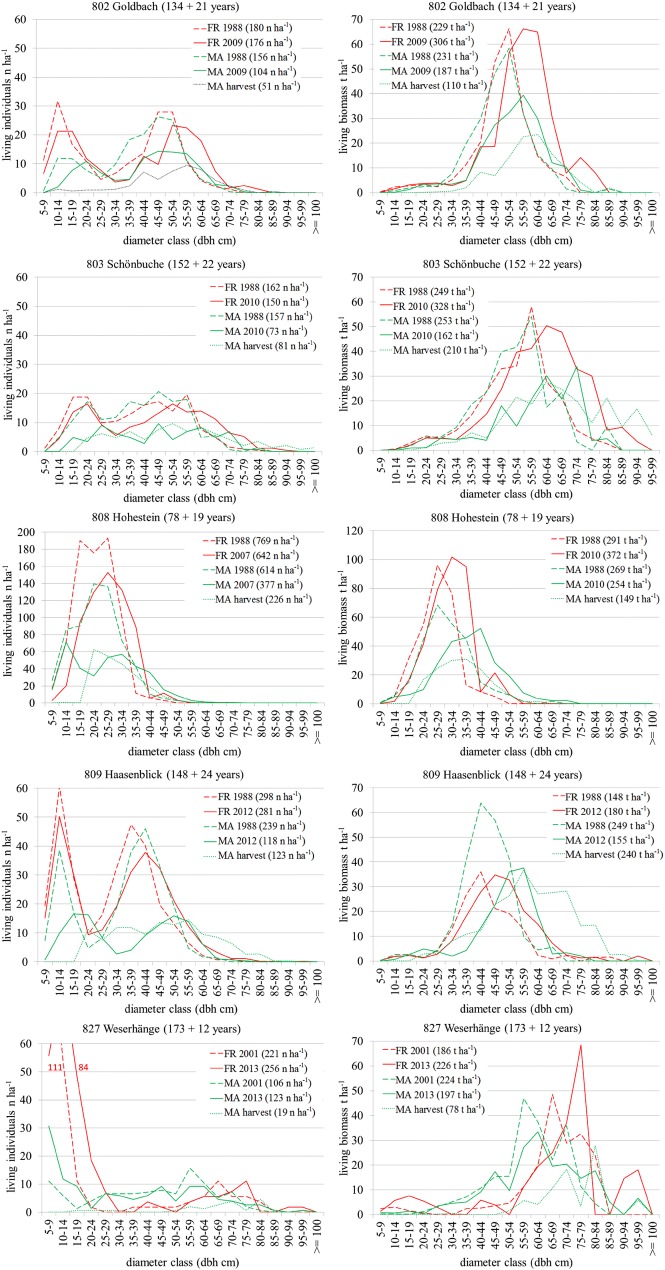
Diameter distributions by the number of individuals and biomass in t ha^−1^ (above- and below-ground living biomass) of the five selected sites at the time of the first and second inventory of the forest reserves (FR) and management areas (MA). The age of the dominating stand at the time of the first inventory is indicated “ + ” the time spans till the second. Individuals and biomass per hectare are indicated in brackets

Especially, 808 Hohestein and 809 Haasenblick reflect noticeable peaks in the diameter distribution around 10 to 35 cm dbh at Hohestein in 1988 (dominating age 78 years ± 3.9 years), and around 10 and between 30 to 50 cm dbh at Haasenblick (dominated by 148 ± 4.2-year-old beech). This is reflected by the volume distribution with distinct peaks at 808 Hohestein in the dbh classes 20–40 cm and 30–60 cm at 809 Haasenblick. 827 Weserhänge, contrary to the other sites, which provides the highest number of individuals at 5 to 15 cm dbh and a dominating stand age of 173 ± 7.9 years. These older trees also dominate the volume distribution in diameter classes above 55 cm dbh (Fig. 1).

Common to all sites is also an expectable progression of the diameter and volume distributions in the forest reserves from the first to the second point in time (red line and broken red line in Fig. 1). In the managed areas, in contrast, harvesting impacts (dotted green line) are recognisable in diameter and volume distribution of the remaining stand (green broken line), most remarkably at 803 Schönbuche and 809 Haasenblick, where individuals of larger diameters have been reduced. The second inventory revealed an additional, overall biomass accumulation (cumulative biomass growth including harvested biomass) after 19 years at 808 Hohestein of about + 34.4% in the forest reserve and about + 56.6% in the management area, at 809 Haasenblick, after 24 years, + 40.1% and + 67.1%, respectively. At 808 Hohestein, the biomass accumulation in the management area has been harvested entirely (removal of 226n ha^−1^ between 20 and 45 cm dbh). In the management area of site 809 Haasenblick, more than the additionally accumulated biomass was harvested; about 27.9% less biomass than 24 years before remained (removal of 123n ha^−1^ between 20 and 75 cm dbh). The management area has obviously been subject to strong thinning and/or harvesting impacts; almost all other species apart from oak have been eliminated. When harvested biomass is included, the cumulative biomass growth in the area under management was 22.2% higher at 808 Hohestein and about 27.0% higher at 809 Haasenblick than in the respective forest reserves.

While at all sites the diameter and biomass distributions of the unmanaged stand moved only slightly to the right (reflecting growing diameters), the distribution in the managed stand was clearly affected by harvests whereby a focus on larger diameter classes cannot be observed. Only at 808 Hohestein, the youngest stand, harvesting focussed on individuals of 20–40 cm dbh. Especially at 803 Schönbuche and 809 Haasenblick, harvests also occurred in the diameter classes larger than 70 cm dbh. Comparable observations, but with lower harvesting intensities, can be made at 802 Goldbach and 827 Weserhänge.

827 Weserhänge was also intensively managed in the past for timber (here till 2001), charcoal production (till the 1960s), and historically for feudal hunting, wood, and pasture. Today, 827 Weserhänge is a mature beech forest in a rather opened structure with some oak (*Quercus robur* L. and *petraea* L.), spruce (*Pikes abies* (L.) H. KARST.), and larch (*Larix decidua* MILL.). A major difference from the other stands is the high number of regeneration and young trees below 20 cm dbh. Both forest reserve and management area of 827 Weserhänge are showing a high number of small trees (diameter classes 5–15 cm dbh) and a fairly even distribution of individuals with diameters between 20 and 70 cm dbh in 2001. Twelve years later, both sites show a high number of advanced regeneration (h > 1.3 m and dbh < 10 cm): 303n ha^−1^ in the forest reserve and 169n ha^−1^ in the management area (Fig. 1).

Cumulative biomass growth (including harvests) increased by 7.9% in the forest reserve of 827 Weserhänge and by 16.4% in the management area. Harvesting activities in the management area reduced other species than beech (i.e., oak) and eliminated spruce; the management area remained with 8.5% more biomass accumulation than the forest reserve.

**Fig. 2:**
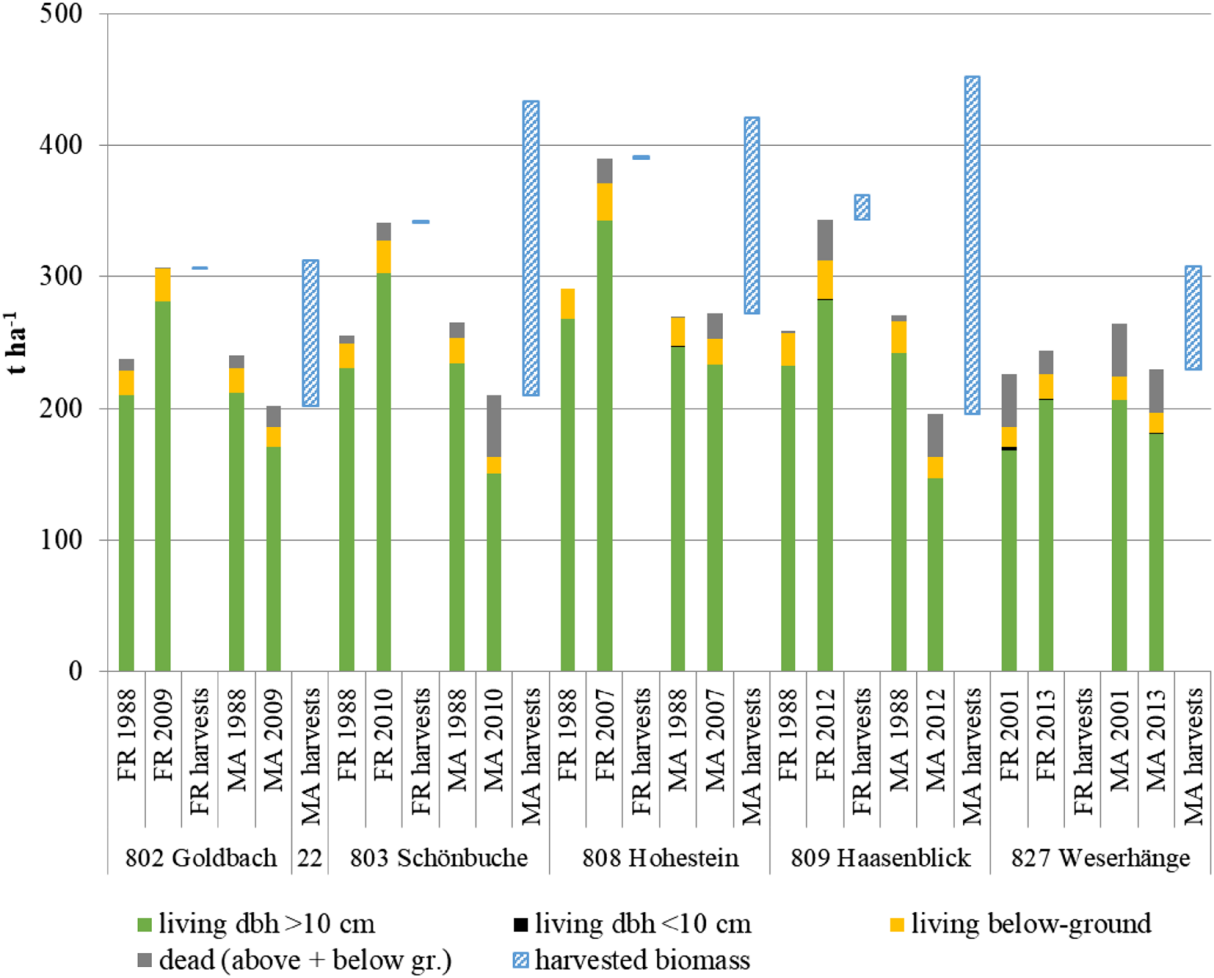
Cumulative biomass growth (above- and below-ground, dead and living), between forest reserves (FR) and management areas (MA) for all species. Beech dominates all stands’ basal areas by 91.4 ± 3.1% (802 Goldbach), 97.9 ± 1.1% (803 Schönbuche), 98.0 ± 2.0% (808 Hohestein), 89.6 ± 9.4% (809 Haasenblick) and 92.9 ± 4.7% (827 Weserhänge). Harvests indicated in forest reserves reflect interventions for safety requirements

Figure 2 illustrates the cumulative biomass growth of the five selected sites and respective compartments for all species. Across all sites, the management areas produced more biomass within the observed time period than the corresponding forest reserves, while at all sites, harvests in the management areas compensated or exceeded biomass increment. An increase of dead wood in the management area is probably caused by the increased number of stumps due to harvesting activities.

Third, while it is obvious that unmanaged forest stands provide higher biomass volume than managed forest stands, the latter are expected to provide a higher periodic biomass accumulation. The relation between increasing periodic biomass accumulation and stand density (basal area) could indicate improved growing conditions. At 809 Haasenblick, for example, beech basal area increased in the forest reserve from 21.5 to 25.3m^2^ ha^−1^, while it was reduced from 22.5to 11.9m^2^ ha^−1^ in the management area. The basal area for oak was more or less maintained (4.2to 4.5m^2^ ha^−1^ in the forest reserve and 3.5 to 3.4m^2^ ha^−1^ in the management area), while other species have been eliminated in the management area. In the second inventory, while in the management area only 51.0% of the forest reserve’s basal area was measured, 74.9% of the forest reserve’s periodic annual increment was recorded. Comparable results are noted for all other sites but 827 Weserhänge. The basal area reduction in the management areas is obviously resulting in an un-proportionally lesser reduction of the annual increment (Table 2). Recognisable also is a higher individual annual biomass increment. A partial removal of larger individuals in the management area of 809 Haasenblick resulted in an increased individual biomass accumulation in the remaining stand by 60.3% for all species and 57.2% for beech. A comparable tendency is noticeable for the other sites.

**Table 2:** Relative difference of management areas compared to respective forest reserves at the time of the second inventory, for the remaining stand (above-ground, living)

Management area	Basal area ha^−1^ (%)		Periodic annual biomass increment ha^−1^ (%)		Periodic individual annual biomass increment (%)	
	All species	Beech	All species	Beech	All species	Beech
802 Goldbach	− 36.9	− 40.0	− 24.8	− 26.8	+ 23.0	+ 22.7
803 Schönbuche	− 50.1	− 50.0	− 36.4	− 36.6	+ 25.1	+ 23.2
808 Hohestein	− 35.4	− 32.2	− 11.9	− 6.0	+ 57.9	+ 56.3
809 Haasenblick	− 49.0	− 52.7	− 25.1	− 29.7	+ 60.3	+ 57.2
827 Weserhänge	− 14.8	− 45.1	− 18.5	− 37.6	+ 91.7	+ 100.2

In total figures, all five forest reserves accumulated an average of 3.7 t (± 1.0 t) biomass ha^−1^ a^−1^, the management areas 6.1 t ha^−1^ a^−1^ (± 2.0 t ha^−1^ a^−1^), including harvested biomass and for all species (Table 3). When harvests are excluded, the management areas lost about 2.0 t (± 1.0 t) biomass ha^−1^ a^−1^. Focussing on beech biomass alone, the forest reserves accumulated 3.3 t ha^−1^ a^−1^ (± 0.9 t ha^−1^ a^−1^), while the management areas accumulated 5.7 t biomass ha^−1^ a^−1^ (± 2.2 t ha^−1^ a^−1^), including harvested biomass, and lost 2.0 t biomass ha^−1^ a^−1^ (± 1.0 t ha^−1^ a^−1^) when harvests are excluded.

**Table 3:** Mean annual biomass accumulation (t ha^−1^ a^−1^, above-and below-ground, dead and living) after 12–24 years

	Biomass (t ha^−1^ a^−1^)			
	All species		Beech	
	Incl. harvest	Excl. harvest	Incl. harvest	Excl. harvest
Forest res. 1	13.6 (± 2.8)	13.6 (± 2.8)	12.5 (± 2.6)	12.5 (± 2.6)
Forest res. 2	17.2 (± 2.6)	17.0 (± 2.7)	15.8 (± 2.4)	15.7 (± 2.4)
Annual diff	3.7 (± 1.0)	3.5 (± 0.9)	3.3 (± 0.9)	3.2 (± 0.8)
%	127.0	125.6	126.2	125.0
Man. area 1	14.2 (± 3.1)	14.2 (± 3.1)	12.7 (± 2.5)	12.7 (± 2.5)
Man. area 2	20.2 (± 2.9)	12.1 (± 3.7)	18.4 (± 2.7)	10.7 (± 3.0)
Annual diff	6.1 (± 2.0)	− 20 (± 1.0)	5.7 (± 2.2)	− 1.9 (± 1.0)
%	142.6	85.6	145.3	84.6
%-points	15.6	− 40.1	19.1	− 40.7

“Forest res. 1” indicates the first inventory of the forest reserve, “forest res. 2” the second, etc.

In relative figures, the management areas provide 15.6% more biomass accumulation by all species, and 19.1% for beech, than the forest reserves when harvests are included.

Some caution must be applied to these results since the statistical analysis provided varying levels of significance by comparing the changes in living biomass volume between the first and second inventory of the forest reserves (compare Table 1). Likewise, it should be considered that such mean biomass values, providing an average of different sites, also comprise different age classes and further potentially varying impacts.

**Fig. 3:**
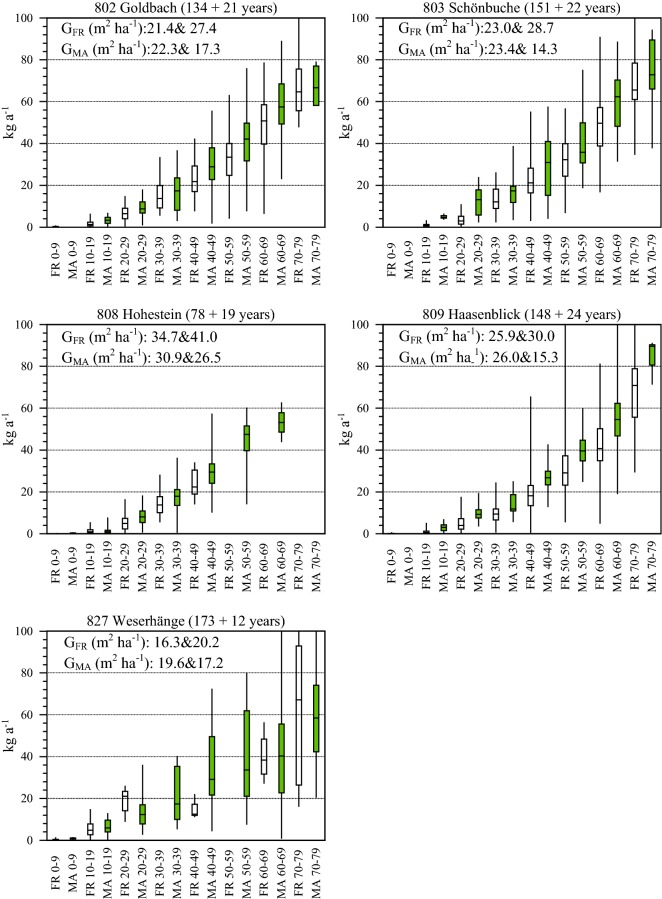
Mean annual increment of individual trees in kg by dbh classes for forest reserves and management areas. Diameter classes larger than 80 cm are excluded due to a low number of individuals. The whiskers describe the range of the respective dataset (min. and max.). Basal areas of forest reserves (G_FR_) and management areas (G_MA_) are provided for the first and second inventory, stand ages and years between the two inventories are indicated in brackets

Fifth, Fig. 3 illustrates the mean periodic increment of individual trees in kg a^−1^ by dbh classes for forest reserves and management areas. Common to all five sites is a larger mean increment in management areas than forest reserves. While the first four sites show higher increment differences in all dbh classes, at 827 Weserhänge larger diameters (≥ 60 cm dbh), the mean increment is comparable in the management area and the forest reserve. It can be concluded that management impacts result in a larger mean increment in almost all sites and diameter classes, presumably as a consequence of thinning and harvesting activities.

Figure 3 also indicates the respective basal area changes to allow a relation to stand density. In the first four sites (802 Goldbach, 803 Schönbuche, 808 Hohestein, and 809 Haasenblick), the basal area is reduced in the management areas while increasing in the forest reserves. Again, 827 Weserhänge provides different results. Here, the basal area is almost maintained in the management area while increasing in the forest reserve.

#### Management impacts on growth dominance structures

The growth-dominance analysis indicates that management leads to more balanced structures in managed than in unmanaged stands. According to the conceptual model from [33] and [34], this reveals a more proportional relation between stand growth and stand mass, resulting from a situation where a suppressing dominance of certain stand compartments is reduced.

**Fig. 4:**
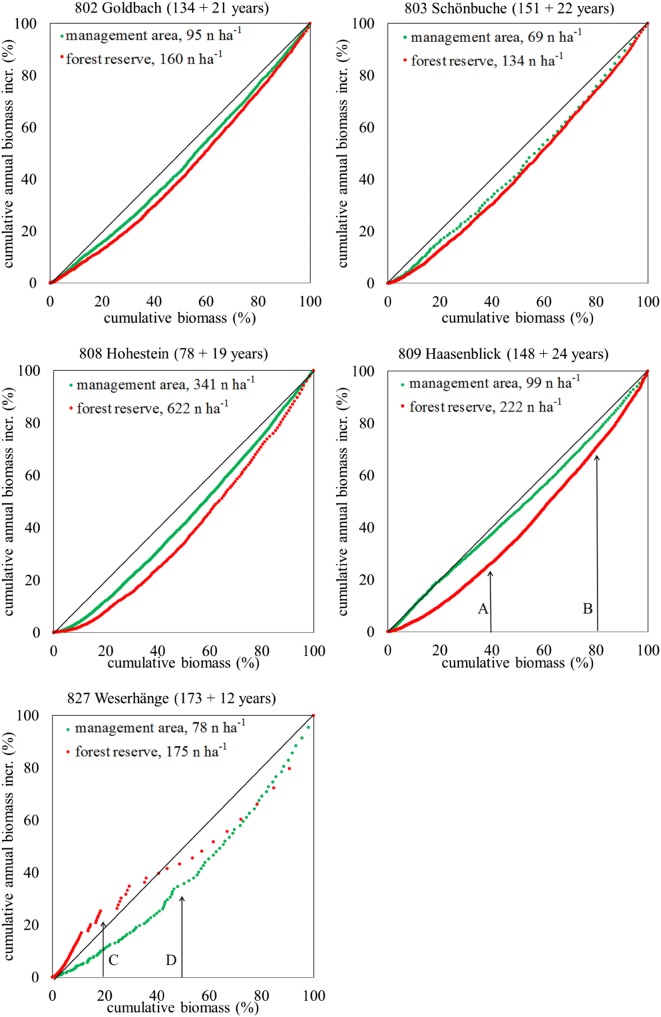
Growth dominance of beech as relationship between cumulative biomass and cumulative biomass increment in forest reserves (red) and respective management areas (green). The indication “A” points to a situation where the smaller trees representing 40% of the cumulative biomass are providing less than 25% of the cumulative biomass increment in the forest reserve, but about 40% of cumulative biomass increment in the management area. At “B” individuals that represent 80% of the management area’s biomass contribute to about 77% to the stand’s biomass increment. At “C” the smaller trees of the forest reserve, representing about 20% of the cumulative biomass, are providing about 30% of the cumulative biomass increment. At “D” the individuals of the management area contributing 50% of the stand’s biomass account for little more than 30% of the stand’s biomass increment

The relative proportionality of the cumulative biomass growth to the cumulative biomass increment of the five sites’ forest reserves and management areas are illustrated in Fig. 4. Following Binkley and Binkley et al*.*, the approximation to the diagonal 1:1 line indicates a situation of low dominance where trees in all dimensions are accounting proportionally to stand growth and stand mass. Comparable at all sites (but 827 Weserhänge) is the observation that the management areas indicate a lower dominance situation than the forest reserves. The first four sites (802 Goldbach, 803 Schönbuche, 808 Hohestein, and 809 Haasenblick) present downwards bent dominance curves. According to Binkley’s growth-dominance model, this describes a situation where larger trees account for a disproportionately large amount of total stand growth, dominate and potentially suppress smaller trees. A closer approximation of the management area’s dominance curves towards the 1:1 diagonal implies a lower dominance situation in management areas than in forest reserves, best recognisable with 808 Hohestein and 809 Haasenblick.

At 809 Haasenblick, for example, all individuals that cumulatively account for 40% of the stand’s biomass in the forest reserve account for about 25% of the stand’s biomass increment. In the respective management area, the lower 40% of the management area’s biomass accounts proportionally for about 40% of the management area’s biomass increment (indication “A” in Fig. 4). While the smaller trees of the forest reserve (cumulative biomass below 50%) represent less than 30% of biomass increment, the larger trees (cumulative biomass larger than 50%) present more than 70% of biomass increment and show an inclining curve; the contribution to the cumulative increment is increasing. These conditions explain increasing growth dominance: Larger trees have gained dominance, increased their use of site resources, and potentially suppressed the growth of smaller trees [34]. In the management area, the dominance curve follows the 1:1 diagonal far closer; only for the larger individuals, the green curve lies slightly below the 1:1 diagonal. Individuals representing 80% of biomass dominance also account less for stand increment than stand biomass but to a smaller extent, about 77% (indication “B” in Fig. 4). A situation where individuals dominate others by mass un-proportionally, with regard to their increment, is obviously resulting in lower increment rates. Figure 3 illustrates, e.g., for 809 Haasenblick far higher mean increment rates in the management area than in the forest reserve. These patterns are, in principle, similar in the first four sites.

Only 827 Weserhänge indicates a different situation again. Here, the smaller individuals of the forest reserve contribute proportionally more to the stand’s biomass increment than to the stand’s biomass. The smaller 20% of the stand’s biomass are providing almost 30% of the cumulative increment (“C” in Fig. 4). In the management area, in contrast, the individuals representing about 50% of the stand’s biomass contribute little more than 30% of the stand`s cumulative biomass increment (“D” in Fig. 4). At both sites, forest reserve and management area of 827 Weserhänge, strong regeneration occurs. While the regeneration seems to dominate the forest reserve next to few large individuals (compare Fig. 1), in the management area, these compartments are more balanced. The rather convex form of a dominance curve in the forest reserve is explained by Binkley et al*.* as a condition of “reverse growth dominance”, which may develop “where the contribution of large trees to total stand growth is less than their proportional biomass, and the curve flattens at the top” [34]. Binkley et al. refer to such conditions, especially to old growth stands. Although 827 Weserhänge cannot be classified as “old growth stand”, comparable dominance conditions seem to occur when the larger trees’ proportional contribution to the stand biomass is less than the smaller ones.

In the management area, this development is dominated by larger trees. Smaller trees contribute proportionally less to the total stand’s cumulative biomass, and the dominance of large trees increases again. In the forest reserve too, the dominance curve approximates the 1:1 diagonal at an early stage and is then dominated by larger individuals that are contributing more to the cumulative mass than increment. Individuals representing 50% of the cumulative biomass contribute more than 70% to the stand’s increment in the management area. In the forest reserve also, 10 large individuals contribute to more than 50% of the cumulative biomass and almost 60% of the cumulative increment (“D” in Fig. 4).

## Discussion

The impact of direct human-induced management was approximated by about 15.6%-point higher cumulative biomass growth in beech-dominated forests in central Germany under BAU. Quite different forest structures and management impacts must be considered. Moreover, an increased biomass accumulation, as result of human-induced management, must first be analysed in relation to the stands’ age classes or the relation of different age classes within the stand. Three sites (802 Goldbach, 803 Schönbuche, and 809 Haasenblick, dominating ages between 134 and 152 years) are dominated by a roughly even distribution of the individuals within all diameter classes below 70 cm dbh, and the respective management areas were subject to intensive harvesting (above increment rates), presumably with a focus on hardwood timber production. 808 Hohestein (dominating age about 78 years) was dominated by diameters below 40 cm dbh, harvesting rates were only slightly above increment rates, and 827 Weserhänge (dominating age about 173 years) by dbh classes below 20 cm. 827 Weserhänge experienced the lowest harvesting rates in the management area.

According to Pretzsch [35], a reduction of the basal area of beech stands can, to a certain extent, result in increased productivity. Joudvalkis et al. [36] demonstrate that significant increase in volume increment is achievable and refer to an enhancement of the crown projection area increment. However, Joudvalkis et al*.* limit this experience to modest thinning intensities and rather young forest stands. This coincides with the observed changes of the mean annual increment in the management areas.

Pretzsch [37] explains that such improved productivity cannot be observed for the whole life cycle but seems to be re-compensated at later stages. Thus, a basal area reduction obviously can, for a certain time period and within distinct age classes, result in increased productivity and provide economic benefits. The observed increased productivity (15.6%-points increased biomass accumulation in the management sites for all species, 19.1%-points for beech alone) can obviously be attributed to a high level of responding ability of *Fagus sylvatica* L. in the observed age classes. This supports Knohl et al*.,* who report that growth rates in old beech forests can remain fairly high at considerable ages [19]. Also, Danescu et al*.* explain an increased productivity in relationship to increased structural diversity [38]. In the same line, Pretzsch indicates canopy structure as driver of stand dynamics [39]. O’Hara and Gersonde explain that especially space occupancy and how growing space is allocated among structural components would affect stand productivity and claim that too little attention would be paid to vertical structures [40]. More precisely, Juchheim et al*.* state that “canopy space filling rather than conventional measures of structural diversity explains productivity of beech stands” and specifically indicate the importance of space-filling of the shaded crown area as indicator of productivity [41]. Following this, it is assumed that management impacts observed during this study facilitate higher structural diversity and thus a higher productivity compared to the forest reserves without management interventions. This assumption is supported by Glathorn et al*.*, who discuss the impact of specific management interventions to the three-dimensional distribution of leaf area in the canopy in primeval and production beech forests [42].

When such different structures and management intensities are compared for identification of the direct human-induced impact by management, it remains questionable whether management as “business as usual” can be defined. Although all sites presented here are managed by the same state forest service by the same management method, harvesting rates are quite different from site to site. The relatively high mean deviation of annual biomass accumulation in the management areas and the obviously large differences between the sites also indicate that a BAU assumption might not be identifiable (compare Table 2). Especially the evaluations from 809 Haasenblick and 827 Weserhänge could support the question to what extent the sites, especially changes in diameter distribution and growth dominance, are comparable at all.

The consideration of the growth dominance curves provides a new approach to answer this question. It is remarkable that all sites but 827 Weserhänge present comparable shapes (compare Fig. 4). In all these sites, the forest reserve’s dominance curve (red) is lower than a 1:1 diagonal. And common to all graphs is the situation that the management area’s curve (green) is closer to the 1:1 diagonal.

From this, the following findings can be derived:

1. All forest reserves (except 827 Weserhänge) are presenting a concave dominance curve where larger trees have the tendency to contribute proportionally more to the stands’ cumulative biomass increment than small ones. This describes an unequal dominance within those stands. Since commonly managed beech stands may have a tendency to form uniform forest stands dominated by large and old individuals (the so-called Hallenwald, “hall-forest”), it can be expected that this dominance of old individuals may increase by age and that concave shapes of the forest reserves’ curves will increase. This development is supposed to be maintained until, e.g., natural causes lead to the collapse of over-aged individuals and provide space and light for younger ones [34].
2. All management areas are presenting a straighter line closer to (and some almost at) the 1:1 diagonal, which is characterising a situation of a more equal dominance structure. At this 1:1 diagonal, the competition between trees is defined to be lower, and each tree’s contribution to the total stand growth is more proportional to its mass [34], obviously a result of management impacts. A presumed harvest of larger individuals reduced the dominance of that compartment of the stand; these changes obviously resulted in increased individual increment rates.

Thus, it is assumed that the management of the investigated beech-dominated forest stands results in a condition where different-aged individuals contribute to the total biomass increment more proportionally to their biomass. This could be intentionally caused as a result of “business as usual”. The management directive for the Hessian state forest promotes a close-to-nature forest management with different-aged individuals for improved stability, diversity, and flexibility so that the forest stands provide an adaptation and development potential that can reflect current and future demands in ecological and economical terms [43]. From the biomass-production point of view, it is the aim to provide high stand volumes of high-quality timber and high increment rates.

When it is assumed that BAU is aiming at high increment rates and the maintenance of relatively high biomass stocks, the application of the growth dominance model could presumably be used to observe respective management impacts, independently of the different age structures and species compositions. Especially the differences of the dominance curves at 827 Weserhänge are remarkable. According to the growth-dominance characteristics, the forest reserve depicts conditions of “reverse growth dominance” [34], while the management impact obviously resulted in a situation comparable to the other sites.

When these conditions are subjected to management and non-management (here, abandonment of wood extraction, thinning regimes, etc.), it can be assumed that the elimination of competing trees and species allowed an improved availability of space, light, and resources to the remaining stand in the management area compared to the forest reserve (compare Binkley et al*.* [34]). The described results of altering diameter distributions and volumes (Figs. 1 and 2), increased increment rates (Table 2 and Fig. 3), and changed growth dominances (Fig. 4) support this assumption. However, it must be considered that a strong basal area reduction would almost automatically lead to more balanced biomass-increment relations. A “more balanced” situation in the growth-dominance model and—following this—an improved productivity of individual trees must not automatically result in improved productivity on stand level (compare Table 2). Here, a relation to the number of individuals is missing. The information value of the growth-dominance model also relies on a minimum number of individuals per ha, which must be acknowledged as a limitation.

In summary, it seems as if forest management has led to a situation where beech trees of all sizes contributed to biomass increment more proportionally, which may explain a higher overall biomass production and higher individual increment rates of the managed compared to the unmanaged stands for all five sites. This is reflecting a high level of responding ability of the observed species. Further, it could be assumed that the impact of management led to a more equally distributed increment along the dimensions of the whole stand compared to non-management. This situation concurs with superior increment rates (almost 16%-points higher in management areas than in forest reserves) and the results illustrated in Fig. 3, where almost all diameter classes provide higher increment rates in the management area than in the forest reserve. Here, management activities and intensive harvesting led to a more efficient overall stand productivity, higher increment, and biomass accumulation.

However, it must again be considered that only a relatively brief time period and distinct age classes are investigated here: It is an important limitation that the observed time spans (12 to 24 years) are relatively short when measured against the stands’ ages. Thus, an overall relative change in % can allow an approximation but not a generalising statement on a superiority of beech forest management. Moreover, when the five observed stands are compared to natural beech forests, e.g., with individual ages of up to 500 years and basal areas of up to 49.5 m^2^ ha^−1^ in the relic virgin beech forest Uholka in the Ukrainian Carpathians, it is obvious that no general statement about a superiority of managed forest stands to natural forests can be allowed; compare [30–32, 44].

## Conclusions

The main objective of this study, the assessment of direct human-induced impacts on forest growth, can be answered with meaningful outcomes. Forest management in the observed beech-dominated forest stands leads to a 15.6% higher cumulative biomass growth compared to stands where management was discontinued and when harvested biomass is considered. The cumulative biomass growth of beech alone is noted as about 19.1% higher in management areas than in adjacent forest reserves. The comparison of the selected forest reserves and adjacent management areas to a larger extent (about 450 ha beech-dominated forests) allows this conclusion for the observed stand age classes.

The assessment enables further conclusions about management impacts on stand structures supporting higher increment rates. Especially the analysis of growth-dominance structures within the assessed sites indicates that forest management resulted in more balanced dominance structures, less competition pressure, and these in higher individual biomass increment. Forest management led to a situation where trees of all sizes contributed to biomass increment more proportionally, which may be explained by potentially improved resource-use efficiency (light, water, nutrients). This is mirrored by higher individual increment rates in all diameter classes above 10 cm dbh in the management areas. It could be concluded that “business as usual” forest management of the assessed management areas aimed at increased hardwood biomass increment combined with the maintenance of high stocks. This reflects the high responding ability of *Fagus sylvatica* L. to management impacts, i.e., density changes.

These results affirm a potential superiority of managed forests to forests where the management was abandoned in terms of biomass accumulation and reveal the impact and effect of the respective interventions. From this, specific recommendations for improved biomass production in uneven aged forest stands could be derived in relation to the observed stand age classes. A further investigation of dominance structures in uneven aged forest could provide more guidance for management interventions – the growth-dominance model could serve as basic concept as demonstrated.

Especially nowadays, while climate change is increasingly affecting the growth of forests and the traditional guidance to determine thinning intensities via yield tables tend to be outdated, it is important to understand how silvicultural interventions affect growth.

The results may support the importance of forest management, especially since the meaning and effect of forest management is contradictorily disputed when managed and unmanaged forests are compared concerning carbon sequestration. Having a system in place that compares representatively managed and unmanaged forests sites to understand better the effects of management changes may be an important prerequisite for accounting for directly human-induced effects more accurately. However, for assessing the full carbon balance of the different treatments, an assumption about the fate of harvested wood is also needed.

## Methods

### Study sites

In 1988, 23 forest reserves were established in the federal state of Hesse, Germany. The sites that all had been managed until 1988 are more or less evenly distributed across the state and comprise an area of 821.5 ha. For almost each of the 23 forest reserves, an adjacent managed “business as usual” (BAU) forest area was designated for research purposes, reflecting similar site and stand conditions and comparable area sizes. Both forest reserves and management areas were inventoried in 1988 and about 20 years later (for some sites, the first inventory took place later). The inventories were carried out by use of permanently marked circular sample plots (diameter: 17.84 m) in a 100 m × 100 m grid [45]. Sampled data used for this study comprise the woody above- and below-ground biomass of standing and lying, living and dead trees. Harvests were documented within the usual harvesting protocols, but for this study, harvests are recorded on the base of the inventory data.

For this study, five beech-dominated (*Fagus sylvatica* L.) forest reserves with adjacent management areas were selected. To facilitate a comparability of the selected stands, only beech-dominated eutrophic and semi-eutrophic sample plots with fair humidity are considered. The following data of different forest reserves and management areas describe pooled samples for each site and time of inventory with no distinct consideration of single sample plots as sub-samples. In total, 211.9 ha of forest reserve and 223.8 ha of management area were selected (overview in Table 4). Other cofounding factors, notable site conditions, are presumably held constant between the paired areas.

**Table 4:** Overview of the selected sites in the federal state of Hesse in central Germany

Forest site number and name	Forest reserve (ha)	Management area (ha)	First inventory (age)	Second inventory
802 Goldbach	31.3	36.9	1988 (133–135)	2009
803 Schönbuche	27.9	26.9	1988 (144–155)	2010
808 Hohestein	26.7	24.4	1988 (73–86)	2007
809 Haasenblick	46.0	41.5	1988 (140–155)	2012
827 Weserhänge	80.0	94.1	2001 (165–186)	2013
Total	211.9	223.8		

The selected sites are dominated by beech forests on sandstone (Hainsimsen-Buchenwald, *Luzulu-Fagetum*) or on alkali-rich and lime soils (Waldgersten-Buchenwald, *Hordelymo-Fagetum*, and Waldmeister Buchenwald, *Galio-Fagetum*). These forest communities are typical for central Germany and have been under intensive utilisation since medieval times [46]. The average annual precipitation in the selected sites on altitudes between 300 and 600 m above sea-level is about 850 mm, the mean annual temperature at ca. 7.7 °C [47]. Secondary podsol sites (soil texture: clay to coarse clay) provide “well to rich nutrient supply” and “well humidity”. General descriptions of all sites are provided by Schmidt and Meyer [48], and publications on changing structures and diversity towards old-growth conditions are provided by Meyer [49] and Hessen-Forst [46].

### Data analysis

Changes in forest biomass between the two inventories for each pair are compared by using allometric equations for living biomass (standing, above- and below-ground), dead biomass (standing/lying, above- and below-ground), and harvested biomass. The following biomass equations of Kändler and Bösch [50] for above- and below-ground biomass, developed for the National Inventory Report (NIR), were used for living and dead trees (Table 5).

**Table 5:** Biomass equations used, exemplified with parameters for beech (*Fagus sylvatica*)

Above-ground biomass (kg), dbh > 10 cm y = b_0_*e^b1^(dbh/(dbh + k1))*e^b2^(d03/(d03 + k2))*h^b3^ y = 0.16787*e^6.25452^*(dbh/(dbh + 11))*e^6.64752^*(d_03_/(d_03_ + 135))*h^0.80745^
Above-ground biomass (kg), dbh < 10 cm and h > 1.3 m y = b_0_ + ((b_s_ − b_0_)/d_s_^2^ + b_3_(dbh – d_s_))*dbh^2^ y = 0.09644 + ((33.22328 – 0.09644)/10 ^2^ + 0.01162*(dbh – 10))*dbh^2^
Below-ground biomass (kg) y = b_0_*dbh^b^ y = 0.018256*dbh^2.321997^
Height function h = 1.3 + 1(a + b/d_1.3_)^−3^ h = 1.3 + 1(0.29397 + 1.76894/d_1.3_)^−3^
Diameter in 30% tree height, d_03_ d_03_ = c_0_*d_1.3_^c1^ d_03_ = 0.84014*d_1.3_^0.9897^

Different to other widely used equations (e.g., the biomass equations from Hochbichler [51] or NW-FVA [52], Kändler and Bösch provide specific parameters for all main species and make use not only of dbh but also of a derived second diameter measured in 30% of the overall tree height (“d_03_”). The evaluations are solely based on woody above- and below-ground biomass of living trees ≥ 7 cm dbh and dead wood ≥ 20 cm at the butt end, using the same equations for living and dead individuals. Stumps and lying dead trees were included as dead-wood. A limitation of the chosen approach is that above-ground biomass of harvested individuals were accounted as completely removed (below-ground biomass was accounted as dead-wood, remaining on-site). Since small branches and parts of the crown are typically left behind in the forests, the estimated biomass removal might be too high. Thus, more biomass than calculated is typically remaining on-site. As a result, dead biomass of the managed forest might be underestimated.

Harvested biomass was calculated by referring to stumps that have been living and standing individuals in the first survey only. The diameter of stumps (recorded in 30 cm above ground) was multiplied by a correction factor of 0.95 to reflect a respective diameter in breast height (130 cm above ground) of the harvested tree. This correction factor was derived from samples collected from forest management areas, which resulted in 10.2% higher diameter at stump height than at breast height (y = 8.9301e^0.0162x^, R^2^ = 0.9411, n = 106). A further consideration of shrinking processes (compare DIN [53] and Sell [54]) added to an estimated 5% smaller dbh at the time of felling than stump diameter 30 cm above ground at the time of the second inventory. Trees that were not recorded in the first stage but in the second and trees that could not be found (neither as standing tree nor as stump) in the second inventory were eliminated from the data set. Regeneration (h < 130 cm) was neglected. Finally, the dataset comprises around 13 000 individuals with two readings each.

The evaluation of biomass changes is made in reference to:

I. The cumulative biomass growth (stand biomass including harvested biomass within the observed time period), as well as
II. In terms of the biomass remaining on site (ignoring harvested biomass).
III. Biomass changes are illustrated according to diameter classes and different pools of above- and below-ground, dead and living biomass for beech and “all” species.
IV. Changes of the mean increment, referring to the observed time period, are also shown by diameter classes.
V. To evaluate the management impact on the stand structure in more detail, growth dominance was determined as a function of stem mass in relation to stem mass increment.

Biomass is referred to carbon in the relationship of 2:1 [55].

The determination of growth dominance is following the conceptual model by Binkley and Binkley et al*. *[33, 34], where four phases of growth dominance in stand development are observable. According to Binkley et al*.* “Growth dominance relates the distribution of growth rates of individual trees within a stand to tree sizes. Stands with large trees that account for a greater share of stand growth than of stand mass exhibit strong growth dominance.” Accordingly, stands with large trees that contribute less to stand growth than to stand mass show reverse growth dominance. Binkley et al*.* established a four-phase model that predicts that forests change from a period of little dominance (phase 1: trees accounting for similar contributions to stand growth and stand mass) to a period of strong growth dominance (phase 2: larger trees account for a disproportionately large amount of total stand growth). Growth dominance then decreases as one or more factors drive a reduction in the growth rate of dominant trees (phase 3), and a condition of “reverse growth dominance” may develop (phase 4) where the contribution of large trees to total stand growth is less than their proportional mass. Figuratively, the curve flattens at the top (Binkley et al*.*).

## Data Availability

The datasets during and/or analysed during the current study available from the corresponding author on reasonable request.

## Abbreviations

GHG: greenhouse gas
LULUCF: Land Use, Land-Use Change and Forestry
BAU: business as usual
NIR: National Inventory Report
